# Supplementary material for: The association between self-reported stress and cardiovascular measures in daily life: A systematic review
Source: PLoS One. 2021 Nov 19;16(11):e0259557. doi: 10.1371/journal.pone.0259557 (PMC8604333; doi:10.1371/journal.pone.0259557)
Supplement: S1 File — (DOCX) [file pone.0259557.s001.docx]

S1 File. Search strategy

*PubMed 6/6/2019:*

(mood[tiab] OR affect[tiab] OR emotion[tiab] OR unpleasant*[tiab] OR hassle*[tiab] OR stress*[tiab] OR distress[tiab] OR (demand[tiab] AND control[tiab]) OR conflict[tiab] OR threat[tiab] OR strain[tiab] OR "negative event*"[tiab] OR "negative social"[tiab]) AND ("holter monitoring"[tiab] OR "heart rate"[tiab] OR electrocardio*[tiab] OR ECG[tiab] OR pulse[tiab] OR cardiovascular[tiab] OR "blood pressure"[tiab] OR “skin response”[tiab] OR "skin conductance"[tiab] OR electrodermal[tiab]) AND ("ambulatory monitoring"[tiab] OR "ambulatory assessment"[tiab] OR "experience sampling"[tiab] OR momentary[tiab] OR diary[tiab] OR diaries[tiab])
