# Supplementary material for: The association between self-reported stress and cardiovascular measures in daily life: A systematic review
Source: PLoS One. 2021 Nov 19;16(11):e0259557. doi: 10.1371/journal.pone.0259557 (PMC8604333; doi:10.1371/journal.pone.0259557)
Supplement: S2 File — (DOCX) [file pone.0259557.s002.docx]

S2 File. Study population characteristics per cardiovascular measure

Studies investigating blood pressure consisted of healthy participants (N = 2,375; 98.2%), individuals with at-risk for cardiovascular diseases (N = 194, 7.5%), or individuals with post-traumatic stress disorder (N = 19; 0.7%). Heart rate studies consisted of healthy participants (N = 1,795; 79.7%), individuals with at-risk for cardiovascular diseases (N = 162; 7.2%), individuals with post-traumatic stress disorder (N = 118; 5.4%), individuals at-risk for psychosis (N = 67; 3.0%), individuals with borderline personality disorder (N = 50; 2.2%), individuals with substance use disorder (N = 40; 1.8%), or individuals with psychosis (N = 20; 0.9%). Heart rate variability studies consisted of healthy participants (N = 716; 71.5%), individuals with cardiovascular diseases (N = 135; 13.5%), individuals with post-traumatic stress disorder (N = 80; 8.0%), individuals with borderline personality disorder (N = 50; 5.0%), or individuals with psychosis (N = 20; 2.0%).
