# Supplementary material for: The association between self-reported stress and cardiovascular measures in daily life: A systematic review
Source: PLoS One. 2021 Nov 19;16(11):e0259557. doi: 10.1371/journal.pone.0259557 (PMC8604333; doi:10.1371/journal.pone.0259557)
Supplement: S1 Table — (DOCX) [file pone.0259557.s005.docx]

S1 Table. Associations based on study methods.

**Associations based on stress measure and study length**

*Systolic and diastolic blood pressure*

|  |  |  | SBP mean | SBP median | DBP mean | DBP median |
| --- | --- | --- | --- | --- | --- | --- |
| Average number of study days for non-significant analyses | | | 1.8 | 1 | 1.9 | 1 |
| Average number of study days for marginally significant analyses (i.e., p<.1) | | | 1.7 | 1 | - | - |
| Average number of study days for significant analyses | | | 2.7 | 1 | 2.9 | 2 |

*Heart rate*

|  | Mean | Median |
| --- | --- | --- |
| Average n days for non-significant analyses | 2.3 | 2 |
| Average n days for marginally significant analyses (i.e., p<.1) | 4 | 4 |
| Average n days for significant analyses | 6 | 2 |

Marginally significant (i.e., *p* < .10)

*Heart rate variability*

|  | Mean | Median |
| --- | --- | --- |
| Average n days for non-significant analyses | 1.9 | 1.5 |
| Average n days for marginally significant analyses (i.e., p<.1) | 2.5 | 2.5 |
| Average n days for significant analyses | 1.6 | 2 |

**Associations based on the sampling technique of self-reported stress**

*Systolic blood pressure*

|  | YES | MARGINAL | NO | TOTAL | YES | MARGINAL | NO |
| --- | --- | --- | --- | --- | --- | --- | --- |
| Random | 2 | 2 | 6 | 10 | 20% | 20% | 60% |
| Fixed | 10 | 2 | 14 | 26 | 38% | 8% | 54% |
| Mixed* | 1 | 0 | 0 | 1 | 100% | 0% | 0% |

* Protocol for self-reported stress used both random and fixed sampling

*Diastolic blood pressure*

|  | YES | MARGINAL | NO | TOTAL | YES | MARGINAL | NO |
| --- | --- | --- | --- | --- | --- | --- | --- |
| Random | 2 | 0 | 11 | 13 | 15% | 0% | 85% |
| Fixed | 8 | 0 | 19 | 27 | 30% | 0% | 70% |
| Mixed* | 1 | 0 | 0 | 1 | 100% | 0% | 0% |

* Protocol for self-reported stress used both random and fixed sampling

*Heart rate*

|  | YES | MARGINAL | NO | TOTAL | YES | MARGINAL | NO |
| --- | --- | --- | --- | --- | --- | --- | --- |
| Random | 4 | 3 | 15 | 22 | 18% | 14% | 68% |
| Fixed | 4 | 1 | 7 | 12 | 33% | 8% | 58% |
| Mixed* | 1 | 0 | 0 | 1 | 100% | 0% | 0% |

* Protocol for self-reported stress used both random and fixed sampling

*Heart rate variability*

|  | YES | MARGINAL | NO | TOTAL | YES | MARGINAL | NO |
| --- | --- | --- | --- | --- | --- | --- | --- |
| Random | 3 | 2 | 15 | 20 | 15% | 10% | 75% |
| Fixed | 4 | 0 | 0 | 4 | 100% | 0% | 0% |

**Associations based on a stress measure and devices**

*Systolic blood pressure*

|  | YES | MARGINAL | NO | TOTAL | YES | MARGINAL | NO |
| --- | --- | --- | --- | --- | --- | --- | --- |
| Spacelabs 90207 | 2 | 1 | 10 | 13 | 15% | 8% | 77% |
| Spacelabs 90217 | 2 | 1 | 5 | 8 | 25% | 13% | 63% |
| Ambulo2400 | 0 | 2 | 3 | 5 | 0% | 40% | 60% |
| DynaPulse5000A | 0 | 0 | 1 | 1 | 0% | 0% | 100% |
| AccutrackerDX | 1 | 0 | 1 | 2 | 50% | 0% | 50% |
| AccutrackerII | 1 | 0 | 0 | 1 | 100% | 0% | 0% |
| Oscar2 | 3 | 0 | 0 | 3 | 100% | 0% | 0% |
| Omron HEM637 | 1 | 0 | 0 | 1 | 100% | 0% | 0% |
| UA-701 | 1 | 0 | 0 | 1 | 100% | 0% | 0% |
| ScottCare | 1 | 0 | 0 | 1 | 100% | 0% | 0% |
| TOTAL | 12 | 4 | 20 | 36 |  |  |  |

*Diastolic blood pressure*

|  | YES | MARGINAL | NO | TOTAL | YES | NO |
| --- | --- | --- | --- | --- | --- | --- |
| Spacelabs 90207 | 3 | 0 | 14 | 17 | 18% | 82% |
| Spacelabs 90217 | 2 | 0 | 6 | 8 | 25% | 75% |
| Ambulo2400 | 0 | 0 | 5 | 5 | 0% | 100% |
| ScottCare | 0 | 0 | 1 | 1 | 0% | 100% |
| DynaPulse5000A | 0 | 0 | 1 | 1 | 0% | 100% |
| Oscar2 | 2 | 0 | 1 | 3 | 67% | 33% |
| AccutrackerDX | 1 | 0 | 1 | 2 | 50% | 50% |
| AccutrackerII | 1 | 0 | 0 | 1 | 100% | 0% |
| Omron HEM637 | 1 | 0 | 0 | 1 | 100% | 0% |
| UA-701 | 1 | 0 | 0 | 1 | 100% | 0% |
| TOTAL | 11 | 0 | 29 | 40 |  |  |

*Heart rate*

|  | YES | MARGINAL | NO | TOTAL | YES | MARGINAL | NO |
| --- | --- | --- | --- | --- | --- | --- | --- |
| Spacelabs 90207 | 1 | 0 | 5 | 6 | 17% | 0% | 83% |
| Spacelabs 90217 | 0 | 0 | 1 | 1 | 0% | 0% | 100% |
| Actiheart | 0 | 0 | 1 | 1 | 0% | 0% | 100% |
| VU-AMS | 1 | 1 | 3 | 5 | 20% | 20% | 60% |
| AccutrackerDX | 0 | 1 | 2 | 3 | 0% | 33% | 67% |
| AccutrackerII | 1 | 0 | 0 | 1 | 100% | 0% | 0% |
| Holter | 0 | 0 | 4 | 4 | 0% | 0% | 100% |
| Ambulo2400 | 0 | 2 | 3 | 5 | 0% | 40% | 60% |
| Movisens | 0 | 0 | 1 | 1 | 0% | 0% | 100% |
| GoldbergeraVR | 1 | 0 | 1 | 2 | 50% | 0% | 50% |
| DynaPulse5000A | 0 | 0 | 1 | 1 | 0% | 0% | 100% |
| OmronHEM637 | 1 | 0 | 0 | 1 | 100% | 0% | 0% |
| VitaportII | 1 | 0 | 0 | 1 | 100% | 0% | 0% |
| AutoSense | 2 | 0 | 0 | 2 | 100% | 0% | 0% |
| UA701 | 1 | 0 | 0 | 1 | 100% | 0% | 0% |
| TOTAL | 9 | 4 | 22 | 35 |  |  |  |

*Heart rate variability*

|  | YES | MARGINAL | NO | TOTAL | YES | MARGINAL | NO |
| --- | --- | --- | --- | --- | --- | --- | --- |
| EcgMove3 | 0 | 0 | 4 | 4 | 0% | 0% | 100% |
| VU-AMS | 0 | 1 | 4 | 5 | 0% | 20% | 80% |
| Holter | 2 | 1 | 3 | 6 | 33% | 17% | 50% |
| EquivitalEQ01LM | 0 | 0 | 1 | 1 | 0% | 0% | 100% |
| Marquette8000 | 1 | 0 | 2 | 3 | 33% | 0% | 67% |
| VitaportII | 0 | 0 | 1 | 1 | 0% | 0% | 100% |
| Zymed | 4 | 0 | 0 | 4 | 100% | 0% | 0% |
| TOTAL | 7 | 2 | 15 | 24 |  |  |  |
