# Supplementary material for: The association between self-reported stress and cardiovascular measures in daily life: A systematic review
Source: PLoS One. 2021 Nov 19;16(11):e0259557. doi: 10.1371/journal.pone.0259557 (PMC8604333; doi:10.1371/journal.pone.0259557)
Supplement: S2 Table — (DOCX) [file pone.0259557.s006.docx]

S2 Table. Methodological quality of the included studies.

|  | Downs and Black Scale domains and items (1–27)* | | | | | | | | | | | | | | | | | | | | | | | | | | |  |  |
| --- | --- | --- | --- | --- | --- | --- | --- | --- | --- | --- | --- | --- | --- | --- | --- | --- | --- | --- | --- | --- | --- | --- | --- | --- | --- | --- | --- | --- | --- |
|  | Reporting | | | | | | | | | | External validity | | | Bias | | | | | | | Confounding | | | | | | Power |  |  |
| Author (year) | 1 | 2 | 3 | 4 | 5 | 6 | 7 | 8 | 9 | 10 | 11 | 12 | 13 | 14 | 15 | 16 | 17 | 18 | 19 | 20 | 21 | 22 | 23 | 24 | 25 | 26 | 27 | Total^α^ | Quality |
| Bacon (2004) | Y | Y | Y | N | Y | Y | Y | N | Y | Y | U | U | N | N | N | Y | Y | Y | Y | Y | N | Y | N | N | N | Y | Y | 17 | Fair |
| Birk (2019) | Y | Y | Y | N | Y | Y | Y | N | Y | Y | U | U | N | N | N | Y | Y | Y | U | Y | Y | Y | N | N | Y | Y | Y | 18 | Fair |
| Buckley (2004) | Y | Y | Y | N | Y | Y | Y | N | Y | N | U | U | N | N | N | Y | Y | Y | U | Y | Y | Y | N | N | Y | U | N | 15 | Fair |
| Carels (2000) | Y | Y | Y | N | Y | Y | Y | N | Y | Y | U | U | N | N | N | Y | Y | Y | Y | Y | N | Y | N | N | Y | Y | Y | 18 | Fair |
| Cornelius (2018) | Y | Y | Y | N | Y | Y | N | N | Y | N | U | U | N | N | N | Y | Y | Y | Y | Y | Y | Y | N | N | Y | Y | Y | 17 | Fair |
| Dennis (2016) | Y | Y | Y | N | Y | Y | Y | N | Y | N | U | U | N | N | N | Y | Y | Y | Y | Y | Y | Y | N | N | Y | Y | Y | 18 | Fair |
| Dennis (2017) | Y | Y | Y | N | Y | Y | Y | N | Y | N | U | U | N | N | N | Y | Y | Y | Y | Y | Y | Y | N | N | Y | Y | Y | 18 | Fair |
| Dennis (2018) | Y | Y | Y | N | Y | Y | Y | N | Y | N | U | U | N | N | N | Y | Y | Y | Y | Y | Y | Y | N | N | Y | Y | Y | 18 | Fair |
| Ebner-Priemer (2008) | Y | Y | Y | N | Y | Y | Y | N | Y | Y | U | U | N | N | N | Y | Y | Y | Y | Y | Y | U | N | N | Y | Y | U | 17 | Fair |
| Edmondson (2015) | Y | Y | Y | N | Y | Y | Y | N | Y | Y | U | U | N | N | N | Y | Y | Y | Y | Y | Y | Y | N | N | Y | Y | Y | 19 | Fair |
| Friedmann (2013) | Y | Y | Y | N | Y | Y | Y | N | Y | Y | U | U | N | N | N | Y | Y | Y | Y | Y | N | U | N | N | Y | Y | N | 15 | Fair |
| Hawkley (2003) | Y | Y | Y | N | Y | Y | Y | N | U | N | U | U | N | N | N | N | Y | Y | Y | Y | N | U | N | N | Y | U | Y | 13 | Poor |
| Ilies (2010) | Y | Y | Y | N | N | Y | Y | N | N | N | U | U | N | N | N | Y | Y | Y | Y | Y | Y | U | N | N | N | N | N | 11 | Poor |
| Johnstron (2016) | Y | Y | Y | N | N | Y | Y | N | Y | N | U | U | N | N | N | Y | Y | Y | Y | Y | Y | U | N | N | N | U | Y | 13 | Poor |
| Kamarck (2018) | Y | Y | Y | N | Y | Y | Y | N | Y | Y | U | U | N | N | N | Y | Y | Y | U | Y | Y | Y | N | N | Y | Y | Y | 18 | Fair |
| Kamarck (1998) | Y | Y | Y | N | Y | Y | Y | N | Y | N | U | U | N | N | N | Y | Y | Y | Y | Y | Y | Y | N | N | Y | Y | Y | 18 | Fair |
| Kennedy (2015) | N | Y | Y | N | N | Y | Y | N | Y | Y | U | U | N | N | N | N | Y | Y | U | Y | Y | Y | N | N | U | N | N | 11 | Poor |
| Kimhy (2010) | Y | Y | Y | N | N | Y | Y | N | Y | Y | U | U | N | N | N | Y | Y | Y | Y | Y | Y | U | N | N | N | Y | N | 14 | Poor |
| Krkovic (2018) | Y | Y | Y | N | N | Y | Y | N | Y | Y | U | U | N | N | N | Y | Y | Y | Y | Y | U | U | N | N | U | Y | Y | 14 | Poor |
| Lehman (2015) | Y | Y | Y | N | Y | Y | N | N | Y | Y | U | U | N | N | N | Y | Y | Y | Y | Y | Y | U | N | N | Y | Y | Y | 17 | Fair |
| Lehman (2010) | Y | Y | Y | N | Y | Y | Y | N | U | Y | U | U | N | N | N | Y | Y | Y | Y | Y | Y | Y | N | N | Y | Y | Y | 18 | Fair |
| Luecken (2009) | Y | Y | Y | N | Y | Y | Y | N | Y | N | U | U | N | N | N | Y | Y | Y | Y | Y | Y | Y | N | N | Y | Y | Y | 18 | Fair |
| Meininger (2004) | Y | Y | Y | N | Y | Y | Y | N | Y | Y | U | U | N | N | N | Y | Y | Y | Y | Y | Y | Y | N | N | Y | Y | Y | 19 | Fair |
| Picot (1999) | Y | Y | Y | N | Y | Y | Y | N | Y | Y | U | U | N | N | N | Y | Y | Y | U | Y | Y | U | N | N | Y | Y | N | 16 | Fair |
| Pieper (2007) | Y | Y | Y | N | Y | Y | Y | Y | Y | Y | U | U | N | N | N | Y | Y | Y | Y | Y | Y | U | N | N | Y | Y | Y | 19 | Fair |
| Pieper (2010) | Y | Y | Y | N | Y | Y | Y | Y | Y | Y | U | U | N | N | N | Y | Y | Y | U | Y | Y | U | N | N | Y | Y | Y | 18 | Fair |
| Pollard (2007) | Y | Y | Y | N | Y | Y | Y | N | Y | Y | U | U | N | N | N | Y | Y | Y | Y | Y | Y | U | N | N | Y | Y | N | 17 | Fair |
| Riediger (2014) | Y | Y | Y | N | Y | Y | Y | Y | Y | Y | U | U | N | N | N | Y | Y | Y | Y | Y | Y | U | N | N | Y | Y | Y | 19 | Fair |
| Schilling (2020) | Y | Y | Y | N | Y | Y | Y | N | Y | Y | Y | Y | N | N | N | Y | Y | Y | Y | Y | Y | Y | N | N | Y | Y | Y | 20 | Good |
| Schmid (2020) | Y | Y | Y | N | Y | Y | Y | N | Y | Y | U | U | N | N | N | Y | Y | Y | Y | Y | Y | U | N | N | Y | Y | Y | 18 | Fair |
| Schwerdtfeger (2019) | Y | Y | Y | N | Y | Y | Y | N | Y | Y | U | U | N | N | N | Y | Y | Y | Y | U | Y | U | N | N | Y | Y | N | 16 | Fair |
| Schwerdtfeger (2014) | Y | Y | Y | N | Y | Y | Y | N | Y | Y | U | U | N | N | N | Y | Y | Y | Y | Y | Y | U | N | N | Y | Y | Y | 18 | Fair |
| Sloan (1994) | N | Y | N | N | N | Y | N | N | Y | Y | U | U | N | N | N | Y | Y | Y | U | Y | U | U | N | N | Y | Y | N | 10 | Poor |
| Thomas (2019) | Y | Y | Y | N | Y | Y | Y | N | Y | Y | U | U | N | N | N | Y | Y | Y | U | Y | Y | Y | N | N | Y | Y | Y | 18 | Fair |
| Tsai (2003) | Y | Y | Y | N | Y | Y | Y | N | Y | N | U | U | N | N | N | Y | Y | Y | U | Y | U | U | N | N | Y | Y | N | 14 | Poor |
| Zawadzki (2016) | Y | Y | Y | N | Y | Y | Y | N | Y | Y | U | U | N | N | N | Y | Y | Y | U | Y | Y | U | N | N | Y | Y | N | 16 | Fair |
| Total for each domain | 34 | 36 | 35 | 0 | 58 | 36 | 33 | 3 | 33 | 25 | 1 | 1 | 0 | 0 | 0 | 34 | 36 | 36 | 26 | 35 | 29 | 17 | 0 | 0 | 30 | 31 | 24 |  |  |

*Downs and Black items are rated as Y = Yes (1), N = No or not applicable (0), U = Unable to determine (0) based on 27 items as follows: 1) Is the hypothesis/aim/objective of the study clearly described?; 2) Are the main outcomes to be measured clearly described in the Introduction or Methods section?; 3) Are the characteristics of the patients included in the study clearly described?; 4) Are the interventions of interest clearly described?; 5) Are the distributions of principal confounders in each group of subjects to be compared clearly described? (this item derived from scoring as follows: 0 = No, 1 = Partial, 2 = Yes); 6) Are the main findings of the study clearly described?; 7) Does the study provide estimates of the random variability in the data for the main outcomes?; 8) Have all important adverse events that may be a consequence of the intervention/study been reported?; 9) Have the characteristics of patients lost to follow‐up been described?; 10) Have actual probability values been reported?; 11) Were the subjects asked to participate in the study representative of the entire population from which they were recruited?; 12) Were those subjects who were prepared to participate representative of the entire population from which they were recruited?; 13) Were the staff, places, and facilities where the patients were treated, representative of the treatment the majority of patients receive?; 14) Was an attempt made to blind study subjects to the intervention they have received?; 15) Was an attempt made to blind those measuring the main outcomes of the intervention?; 16) If any of the results of the study were based on “data dredging”, was this made clear?; 17) In trials and cohort studies, do the analyses adjust for different lengths of follow‐up of patients, or in case‐control studies, is the time period between the intervention and outcome the same for cases and controls?; 18) Were the statistical tests used to assess the main outcomes appropriate?; 19) Was compliance with the intervention/s reliable?; 20) Were the main outcome measures used accurate (valid and reliable)?; 21) Were the patients in different intervention groups (trials and cohort studies) or were the cases and controls (case‐control studies) recruited from the same population?; 22) Were study subjects in different intervention groups (trials and cohort studies) or were the cases and controls (case‐control studies) recruited over the same period of time?; 23) Were study subjects randomised to intervention groups?; Was the randomised intervention assignment concealed from both patients and health care staff until recruitment was complete and irrevocable?; 24) Was the randomised intervention assignment concealed from bothpatients and health care staff until recruitment was complete and irrevocable?; 25) Was there adequate adjustment for confounding in the analyses from which the main findings were drawn?; 26) Were losses of patients to follow‐up taken into account?; 27) Did the study have sufficient power to detect a clinically important effect where the probability value for a difference being due to chance is less than 5%?

^α^  Downs and Black scores, ranging from 26–28 (excellent), 20–25 (good), 15–19 (fair), and ≤ 14 (poor)
