# Supplementary material for: The association between self-reported stress and cardiovascular measures in daily life: A systematic review
Source: PLoS One. 2021 Nov 19;16(11):e0259557. doi: 10.1371/journal.pone.0259557 (PMC8604333; doi:10.1371/journal.pone.0259557)
Supplement: Attachment — Submitted filename: Response to Reviewers.docx [file pone.0259557.s008.docx]

Below we have listed all the comments with a point-by-point response giving specific details as to how we addressed each comment.

Comments from the editor:

1. Please ensure that your manuscript meets PLOS ONE's style requirements, including those for file naming. The PLOS ONE style templates can be found at

https://journals.plos.org/plosone/s/file?id=wjVg/PLOSOne_formatting_sample_main_body.pdf and

https://journals.plos.org/plosone/s/file?id=ba62/PLOSOne_formatting_sample_title_authors_affiliations.pdf

**Response:** We have followed the style templates and revised the manuscript accordingly.

2. Please review your reference list to ensure that it is complete and correct. If you have cited papers that have been retracted, please include the rationale for doing so in the manuscript text, or remove these references and replace them with relevant current references. Any changes to the reference list should be mentioned in the rebuttal letter that accompanies your revised manuscript. If you need to cite a retracted article, indicate the article’s retracted status in the References list and also include a citation and full reference for the retraction notice.

**Response:** We have revised the reference list. Two studies have added during the revision phase [33, 34] due to the study quality assessment.

3. The PRISMA checklist requires that the quality or risk of bias of the included studies is assessed. Furthermore, conclusions in a systematic review or meta-analysis should be related to the quality of the included publications. In light of this concern we would be grateful if you could please update your systematic review to include a formal quality assessment. Please ensure that your quality assessment is suitable for the study type included in your systematic review. Please also note that reporting checklists (e.g. STROBE) are not suitable for use as quality assessment tools.

**Response:** We have revised this review by conducting a methodological quality assessment using the Downs and Blacks Scale. Please see revised sections in the Materials and Methods (on page 8), Results (on page 25), and Discussion (on pages 33–34).

4. We note that you have indicated that data from this study are available upon request. PLOS only allows data to be available upon request if there are legal or ethical restrictions on sharing data publicly. For more information on unacceptable data access restrictions, please see http://journals.plos.org/plosone/s/data-availability#loc-unacceptable-data-access-restrictions.

In your revised cover letter, please address the following prompts:

a) If there are ethical or legal restrictions on sharing a de-identified data set, please explain them in detail (e.g., data contain potentially sensitive information, data are owned by a third-party organization, etc.) and who has imposed them (e.g., an ethics committee). Please also provide contact information for a data access committee, ethics committee, or other institutional body to which data requests may be sent.

b) If there are no restrictions, please upload the minimal anonymized data set necessary to replicate your study findings as either Supporting Information files or to a stable, public repository and provide us with the relevant URLs, DOIs, or accession numbers. For a list of acceptable repositories, please see http://journals.plos.org/plosone/s/data-availability#loc-recommended-repositories.

We will update your Data Availability statement on your behalf to reflect the information you provide.

**Response:** We have added all relevant data files as supporting files for one excel file (S5 File) and also provided the extraction data (S8 File).

5. Please include captions for ALL your Supporting Information files at the end of your manuscript, and update any in-text citations to match accordingly. Please see our Supporting Information guidelines for more information: http://journals.plos.org/plosone/s/supporting-information.

**Response:** We have followed the Supporting Information guidelines and revised the manuscript accordingly.

Comments from the reviewers:

**Reviewer #1:** This is an interesting and relevant overview of the literature on associations between self-reported stress and selected cardiovascular measures. As with all reviews, the outcome is dependent on a variety of choices made by the authors – some might be debatable (such as the focus on at least one day of assessments), but as long as these choices are justified, the end result stands on a relatively firm basis. I have only a few suggestions on how to further improve the paper:

1. The main query I have is that that I would want to ask the authors to put together a brief list of recommendations at the end of their discussion. I think they provide a clear-cut discussion on their findings, and it becomes quite clear what the limitations in the literature are, but at the end the reader is left wondering what to take from the paper for his or her own research. What is, e.g., an ideal segment for the association between self-reported stress and HRV? What is the ideal statistical approach based on which data characteristics? What is the ideal time-lag between self-reported stress and selected measures? Just a few questions I had.

**Response:** This is a very good suggestion. Although it is difficult to provide strong suggestions for all points the referee suggests based on the results of the current review, we have changed the title of the final paragraph to read “Conclusions and recommendations” and added some recommendations based on our findings. This can be found on page 35.

“Therefore, based on this review, we recommend researchers to use the following when investigating the association between self-reported stress and cardiovascular measures:

-	Perceived stress or high-arousal NA self-report measures

-	High-quality wearable sensors that have been validated in ambulatory settings

-	Time windows of max 30 minutes prior to self-report for the calculation of continuously assessed cardiovascular measures

-	Study periods of at least 6 days with multiple assessments per day to capture enough variability in both measures

-	Multilevel modelling for the statistical analyses”

2. The main variables in this review should probably be justified a little more in detail. The authors start with an overview of the biological stress systems, but I don´t think this is of great relevance, when in the end they are only focusing on a few selected cardiovascular measures. They could simply skip to the role of the cardiovascular system and its role in the overall stress process. In this context, it should be noted that there are literally hundreds of ESM studies which measured cortisol, so the statement on page 4 regarding HPA axis markers is not correct.

**Response:** Although we do agree with the referee that it is unnecessary to mention the HPA axis in the introduction, our intention was to review studies investigating ANS measures (ie including skin conductance). As we only found out after our search that there were not enough studies that looked at the association between skin conductance and self-reported stress, we chose to focus on cardiovascular measures only. In our opinion, altering the introduction accordingly would not be in line with our procedures. We therefore deleted the two sentences on the HPA axis on page 4, but left the motivation to investigate the ANS intact. We hope the referee agrees with this decision.

3. I cannot agree that laboratory studies are showing heterogeneous results due to a lack of ecological validity. If one wants to follow the stress appraisal model by Lazarus (and most studies seem to do to some degree), then it doesn´t matter where the stressor takes place. The authors could leave out that statement and still have enough to justify their focus on ESM studies.

**Response:** We fully agree with the referee that the stress system does not differentiate between a laboratory stress task or a daily stressor. However, depending on the nature of the stressor, we do believe the stress response differs between different instances. The very abrupt on- and offset of stressor exposure in the lab, where often anticipatory stress is tried to be kept to a minimum by inclusion of a baseline period, is arguably not comparable to typical daily-life stressors such as work stress, social stressors, ruminative thoughts, etc. that have much less clear start- and endpoints. In that sense, the stress response that we typically observe in the lab probably does not reflect stress system activity in daily life. However, we do agree with the referee that we cannot conclude that this is the reason for heterogeneous results from lab tasks and have therefore deleted this sentence on page 5.

4. I agree that it is difficult to compare studies due to the wide variety of statistical approaches. One of the conclusions could be that it will be of importance to follow-up with a meta-analysis, which would allow for tackling the main research question using one unified statistical approach.

**Response:** This is a very good suggestion and we have added the following sentence to our discussion on page 34.

“Although most studies used MLM, the large diversity between models prevented us from conducting meta-analyses based on the reported values of the associations per stress measure. Therefore, an accurate estimate of the strength of the association could not be provided. Future investigations should put in effort to mirror the statistical models and methods previously used to estimate the association between self-reported and cardiovascular measures of stress so that meta-analyses can be conducted.”

**Reviewer #2:** This systematic review evaluated the evidence for studies that have assessed daily stress using ambulatory methods and associated ratings of self-reported stress with cardiovascular measures. The most interesting point is that it focuses on daily stress. I agree that the results from laboratory setting studies using standardized stress task are different from those measured in the real-life stressful condition. This paper is interesting throughout, but I have a few minor comments.

1. One of the aims of this review is to investigate an association between acute stress and physiological measures. It would be interesting if the analysis could be limited to only those studies that assessed acute stress.

**Response:** We agree with the referee that looking at associations for acute stress only would be very interesting. However, it is, using these data, impossible to disentangle acute from chronic stress. Therefore, to prevent confusion, we have removed the word “acute” from our aims paragraph on page 5.

2. P33 line 3: “One possible explanation for sample differences is that patients and individuals at risk may experience more stress during the day, providing for more variability in the data and hence increase the likelihood of finding an association.” Does this mean that the patients and individuals at risk are more frequently exposed to chronic stress, or does it mean that they are more sensitive to stressors?

**Response:** Good point. We meant overall more fluctuations in their stress levels due to both more exposure to stressors and increased sensitivity to stress, as only higher overall levels of stress would not result in more fluctuations throughout the day. We have clarified this as follows:

“One possible explanation for sample differences is that patients and individuals at risk may experience more fluctuations in their stress levels during the day, providing for more variability in the data and hence increase the likelihood of finding an association.”

3. P34 line2: Validation of BP monitoring device evaluates the accuracy of BP values measured by BP monitoring devices compared with those measured by mercury sphygmomanometers, that is, Spacelabs 90207 is a validated device for monitoring BP.

**Response:** We agree with the reviewer. However, we also believe that we have made this point very clear in our manuscript. In the line the referee refers to, we explicitly state that Spacelabs 90207 indeed is a valid tool for BP monitoring. We do feel that we have to mention the limitations that are described in the literature regarding the device. We hope the referee agrees that our current phrasing accurately describes both.
